# Ten Simple Rules for avoiding and resolving conflicts with your colleagues

**DOI:** 10.1371/journal.pcbi.1006708

**Published:** 2019-01-18

**Authors:** Fran Lewitter, Philip E. Bourne, Teresa K. Attwood

**Affiliations:** 1 Whitehead Institute for Biomedical Research, Cambridge, Massachusetts, United States of America; 2 Data Science Institute & Department of Biomedical Engineering, University of Virginia, Charlottesville, Virginia, United States of America; 3 School of Computer Science, The University of Manchester, Manchester, United Kingdom; Dassault Systemes BIOVIA, UNITED STATES

During the course of our personal and professional lives, we spend a significant amount of time communicating with others. In fact, communication is one of the most important, but possibly also one of the hardest, things we do, having the power to bring individuals and communities together or create divisions. Getting it right is therefore crucial.

Modern technologies have had a significant impact on the ways in which we are now able to communicate, allowing us to share our thoughts with colleagues, family, or friends at the click of a button. But communicating more quickly does not always result in better communication—the technologies we use often divorce us from the visual clues that are so crucial to understanding each other’s true meaning and make it easy to misinterpret each other’s real intentions. For this reason, our interactions can sometimes be unexpectedly difficult or can go unaccountably wrong.

Given that communication is vital to the health and productivity of relationships, how can we best make our interactions work, and how can we resolve situations when they arise? The following are 10 simple rules based on our experience that we hope will help. Many of these rules can apply to the kinds of communication we may have with colleagues, family, or friends. However, we focus this article on the professional environment: we begin with suggestions to help avoid disagreements or to help stop them turning into serious conflicts; we then reflect on steps that might help to resolve situations that have become confrontational.

Most interactions with colleagues are cordial and are working towards a common goal. Sometimes, however, because of differing views, misinterpretation of something said, or just because you’re having a bad day, communications can go awry and become heated; from this point, without resolution, awkward situations can quickly escalate. Practicing effective communication skills before a confrontation arises, or during a confrontation, is the topic of this article. For more general ideas about engaging in successful collaborations, see [1]. To delve further into the area of conflict management in the work environment, see [2, 3]. To keep this contribution manageable, we have confined ourselves to peer-to-peer communication and not considered a larger ecosystem of interactions in which conflict occurs. We feel that is a separate contribution that should be written.

## Rule 1: Always treat people with equality and respect

Whether interacting with your peers or not, treat people courteously. Don’t prejudge individuals based on their rank or perceived academic abilities, or worse, their gender, race, or sexual orientation. Be polite, and treat everyone equally and fairly.

## Rule 2: Seriously consider and respect others’ views

You may not always agree with a colleague’s views, but remember, your colleagues may have different and valuable perspectives based on their past experience; moreover, based on their cultural background, they may have different sensitivities and differences in communication style that go far beyond language differences. Don’t be dismissive of their ideas or values, and contemplate any cultural aspects that should be considered. Remember that the primary reason for interacting with your colleague was to achieve a common goal. Always keep that in mind, and try to work on ways to successfully reach that goal together.

## Rule 3: If you disagree with someone, say so and explain why

During the course of a discussion, you may find that you disagree—perhaps quite strongly—with a colleague. If this happens, remain calm and professional; don’t let anger or resentment build up. In short, communicate calmly and early to prevent confrontation. It’s important to address points of friction as they arise. Be straightforward about what concerns you or what you object to. Give reasons for your point of view—state the facts, not your opinions. In trying to defuse a situation, or to prevent an impasse, it may be helpful to include additional people in the conversation; to be constructive, you should also try to propose a solution to your disagreement.

## Rule 4: Make sure you are on the same page

It is particularly easy to misinterpret communications when discussions are conducted via email or another asynchronous medium. If, because you’re busy, your communications are short and abrupt, they may just come across as rude; on the other hand, if your messages are long and emphatic, they may seem rather dogmatic. To reduce the chance of misinterpretation and to ensure that what you mean to convey isn’t lost, it’s important to be clear in your written communication and to choose your words carefully—be particularly aware of the vocabulary and grammar you use. This is especially important if the other person is not a native speaker of the language you’re using and may miss nuances in what you are saying. During a confrontation, backing up any written communication with a verbal follow up, however hard, is advisable.

## Rule 5: Pause before you press “Send”

Whether you are writing or speaking to a colleague, think before you do so. If you’re upset and plan to send an email, it is advisable to write a draft and put it aside for a while. Sleep on it! Take time to ensure that the content of the message is really what you want to convey and that it is measured in tone and objective (as mentioned in Rule 4, email communication can very easily be misinterpreted). The same goes for verbal communication. If you are irritated or frustrated, take time to compose yourself and to formulate rational arguments before speaking to your colleague. In our experience, this is the most likely way to prevent an escalation in the situation.

## Rule 6: Apologize when you do something wrong

We all make mistakes—a sharp word in a meeting, an email sent in haste, a spontaneous tweet. If you have had a disagreement about an issue or treated someone disrespectfully, there’s nothing wrong (but everything right) in offering a sincere apology, preferably in writing. Whether you want to continue working with the person you wronged or not, it’s best to admit you erred. This is more likely to earn you respect from your colleagues, and in the long run, it will be respect that goes a long way to define you as a scientist [4].

## Rule 7: Engage in an honest nonconfrontational dialogue

If a disagreement has escalated into a conflict, distance yourself from your emotions and document the conflict: note what was said or done to you, when and where, and how it made you feel. Having a written record is extremely useful. Ask to have a conversation with your colleague (whether face-to-face, by telephone, Skype, etc.). Agree on the issue to be discussed; it may be helpful to provide a concise summary to avoid misconceptions. Present your position clearly and listen carefully to the response. Ask for clarification if you don’t understand what is said to you. Don’t let emotions enter into the discussion; avoid raising your voice. If you feel confident, calm, and safe enough to do so, address the behavior. Don’t give your colleague permission to bully you. Let him or her know that the behavior is offensive or, at the very least, making you uncomfortable. If possible, ask where the behavior has come from; try to get to the real issue and help your colleague resolve his or her anger.

## Rule 8: Know who to turn to if you need impartial advice

If you find yourself being confronted by a colleague who uses inappropriate language or who makes you feel threatened, don’t reply in kind. Empower yourself by finding out your organization’s policy on bullying and harassment (you may be protected by their policies or code of conduct) and making yourself aware of the law in your country regarding bullying and harassment in the workplace (in many countries, bullying or offensive, intimidating, and/or abusive behavior of colleagues toward you may actually be unlawful). Take action by reporting the behavior to someone outside the situation—make the impact of the behavior, in terms of how it made you feel, very clear. If you and your colleague are from the same department, the department head may be able to offer advice to help resolve the conflict. However, this option needs to be considered in light of the individuals involved. If not approached carefully, it may backfire and cause further resentment from your colleague, escalating rather than defusing the situation. In this case, you may need to go to a higher level individual outside your department. Many institutions and organizations have ombudspersons to handle conflicts that get out of hand. Find out what your institution or organization offers. Alternatively, you may have an impartial mentor or colleague to whom you may turn for advice, but if a professional skilled in dealing with conflict is available, that is preferred.

## Rule 9: Know when it’s worth fighting for your point of view

There’s an old saying: “Don’t beat your head against the wall. If you do, it feels much better when you stop.” If you are in an argument that is not moving towards resolution, walk away. In the best of circumstances, it is desirable that you and your colleague can simply agree to disagree and move on to other issues, still maintaining mutual respect.

## Rule 10: If the problem is unresolvable, distance yourself

Unfortunately, not all conflicts can be resolved to everyone’s liking. It may be necessary at some point to distance yourself from the source of the problem. That may include resigning from a position, leaving a research project, or shifting your focus to other activities—a sad situation to be in but one that is preferable to continuing to endure an unpleasant environment.

Like it or not, you will encounter a spectrum of conflicts throughout your scientific career. Those range from disagreements as part of the daily scientific discourse—those are what drive science forward—to the unpleasant, in which personalities and associated emotions clash in disagreeable ways. How you handle the conflict spectrum will go a long way to defining you as a scientist. It's a small and intertwined community. What goes around comes around. Act appropriately at all times with, we hope, these rules as your guide.
